# Microstructures Manufactured in Diamond by Use of Laser Micromachining

**DOI:** 10.3390/ma13051199

**Published:** 2020-03-06

**Authors:** Mariusz Dudek, Adam Rosowski, Marcin Kozanecki, Malwina Jaszczak, Witold Szymański, Martin Sharp, Anna Karczemska

**Affiliations:** 1 Institute of Materials Science and Engineering, Lodz University of Technology, 1/15 Stefanowskiego Street, 90-924 Lodz, Poland; mariusz.dudek@p.lodz.pl (M.D.); witold.szymanski@p.lodz.pl (W.S.); 2 SPI Lasers, 3 Wellington Park, Tollbar Way, Hedge End, Southampton, Hampshire SO30 2QU, UK; adam.rosowski@spilasers.com; 3 Institute for Manufacturing, University of Cambridge, 17 Charles Babbage Road, Cambridge CB3 0FS, UK; 4 Department of Molecular Physics, Lodz University of Technology, 116 Zeromskiego Street, 90-924 Lodz, Poland; marcin.kozanecki@p.lodz.pl; 5 Department of Mechanical Engineering, Informatics and Chemistry of Polymer Materials, Lodz University of Technology, 116 Zeromskiego Street, 90-924 Lodz, Poland; malwina.jaszczak@p.lodz.pl; 6 General Engineering Research Institute, Liverpool John Moores University, Byrom Street, Liverpool L3 3AF, UK; m.sharp@ljmu.ac.uk; 7 Institute of Turbomachinery, Lodz University of Technology, 219/223 Wolczanska Street, 90-924 Lodz, Poland

**Keywords:** polycrystalline diamond, laser microprocessing, microstructures

## Abstract

Different microstructures were created on the surface of a polycrystalline diamond plate (obtained by microwave plasma-enhanced chemical vapor deposition—MW PECVD process) by use of a nanosecond pulsed DPSS (diode pumped solid state) laser with a 355 nm wavelength and a galvanometer scanning system. Different average powers (5 to 11 W), scanning speeds (50 to 400 mm/s) and scan line spacings (“hatch spacing”) (5 to 20 µm) were applied. The microstructures were then examined using scanning electron microscopy, confocal microscopy and Raman spectroscopy techniques. Microstructures exhibiting excellent geometry were obtained. The precise geometries of the microstructures, exhibiting good perpendicularity, deep channels and smooth surfaces show that the laser microprocessing can be applied in manufacturing diamond microfluidic devices. Raman spectra show small differences depending on the process parameters used. In some cases, the diamond band (at 1332 cm^−1^) after laser modification of material is only slightly wider and shifted, but with no additional peaks, indicating that the diamond is almost not changed after laser interaction. Some parameters did show that the modification of material had occurred and additional peaks in Raman spectra (typical for low-quality chemical vapor deposition CVD diamond) appeared, indicating the growing disorder of material or manufacturing of the new carbon phase.

## 1. Introduction

Diamond can be considered as a very interesting material for biomedical applications, because of its excellent properties such as the highest known thermal conductivity, biocompatibility, chemical inertness and transparency to light. It is particularly interesting for microfluidic devices manufacturing [1,2]. Currently, many potential applications of diamond are limited by the difficulties in its processing.

One of the most interesting ways of shaping diamond is with the use of laser micromachining. The modification of diamond at very specific points of the sample gives the possibility of new applications. The interaction of the diamond with the laser light can change the physical and mechanical properties in specific places and in specific ways [3,4,5,6,7,8,9,10,11,12]. Cappelli et al. [3] used an ArF excimer nanosecond laser (193 nm wavelength) to smooth the diamond surface and noticed the formation of a thin (<1 µm) glassy amorphous carbon layer, with the Raman bands at 1359 cm^−1^ and at 1589 cm^−1^. This layer was responsible for some new surface properties of the film, such as very high electrical conductivity, higher reflectivity and low photoluminescence. The formation of graphitic-like layers on the surface of diamond by the evaporative ablation process with a high material removal rate (>10 nm pulse^−1^) [12,13], leading to a decrease in laser beam energy density in the laser-irradiated region, can finally stop the process. 

A great possibility of the laser modification of diamond surface is the micromachining of three-dimensional structures, which can help in the development of diamond-based photonic devices and shows the perspectives in the field of Microelectromechanical Systems (MEMS), microfluidics and biophysics [9,14]. The analysis of the nanoablation process of diamond indicates that the photoreaction occurs directly between the surface-layer atoms and adsorbed molecules—one atomic layer of material (*a* = 0.178 nm—the average interatomic distance in the diamond lattice) is generally removed by photoetching during a pulse [10,13]. This indicates that a complementary way to improve the nanoablation processing efficiency is to increase the laser pulse repetition rate. Some more signs of the new ideas and applications can be found in the literature. Nano-pattering of diamond surfaces in high resolution (single atoms scale) was created by the graphite-free laser etching of diamond using 266 nm laser pulses [15,16]. Recently, Ding et al. [17] have shown that diamond films modified with laser ablation could be developed into a microwave attenuation material by obtaining the graphite fibers within diamond. Other applications, which have been implemented recently, are described in the review article by Truccchi at al [18]. The microstructuring and nanostructuring of diamond thanks to ultrashort laser pulses allows modifications of optical and electronic properties of this interesting material; for instance, patterned “black diamod” is an example for future optoelectronics applications.

In the present work, the formation of microstructures in bulk polycrystalline diamond by use of laser (355 nm, 50 kHz) micromachining is investigated. In order to evaluate the effect of diamond modification, several investigations were carried out; by Raman spectroscopy, scanning electron microscopy (SEM) and confocal laser scanning microscopy (CLSM).

## 2. Materials and Methods 

**Figure 1:**
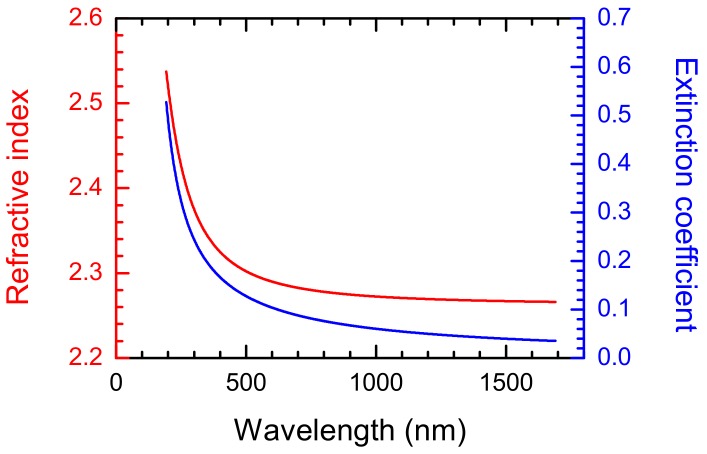
Optical constants of the MW PECVD polycrystalline diamond plate.

A polycrystalline diamond plate with a thickness of 530 μm was obtained with use of a microwave plasma-enhanced chemical vapor deposition (MW PECVD) system DF-100 (Russian Academy of Science, Moscow, Russia) using CH_4_/H_2_ gas mixture [1,19]. The following conditions have been used: microwave power 3.6 kW, methane content 2%, total flow rate 800 sccm, pressure 87 Torr, substrate temperature 820 °C. Before the deposition, the Si substrate was seeded with suspension of a detonation nanodiamond in ethanol by a treatment in an ultrasonic bath. The growth rate of diamond was 1 µm/h. The diamond plate possesses the following properties: hardness 85.1 ± 10.2 GPa, Young’s modulus 1114.5 ± 183.8 GPa, thermal conductivity 1040 / 1280 Wm^−1^K^−1^, and optical constants (characterized by a variable angle spectroscopic ellipsometer—RC2, J.A. Woollam Co. Lincoln, NE, USA) as shown in Figure 1. 

Diamond micromachining was performed using a diode pumped solid state (DPSS) laser (Coherent AVIA, Santa Clara, CA, USA) which is characterized by wavelength—355 nm, pulse repetition rate—50 kHz, average power—up to 14 W, and pulse duration in the range of 25/35 ns (varied with pulse frequency and average power). The UV wavelength is very suitable for the samples due to its high absorption for UV light (Figure 1). 

**Figure 2:**
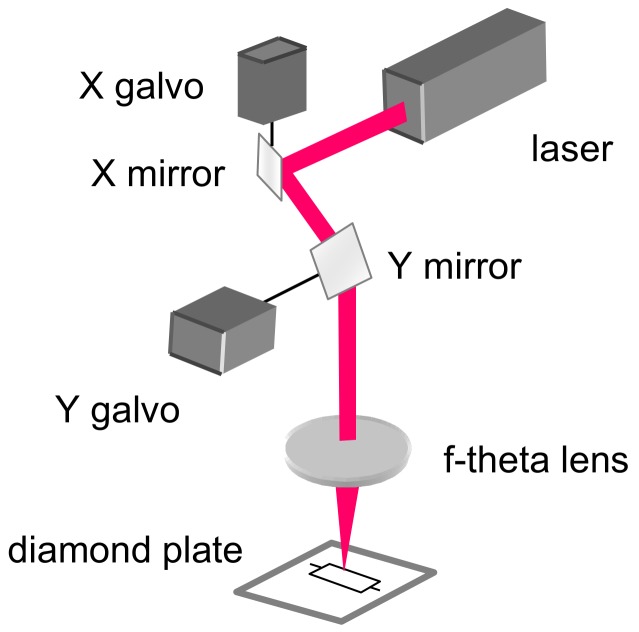
Scanner system.

**Figure 3:**
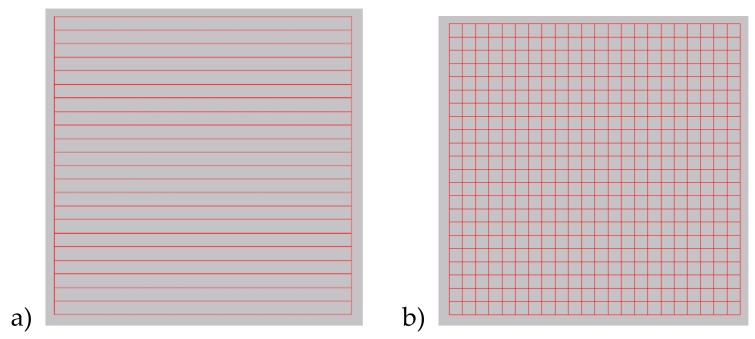
Hatching technique: (**a**) single pattern; (**b**) double-pattern (0^o^ and 90^o^).

**Figure 4:**
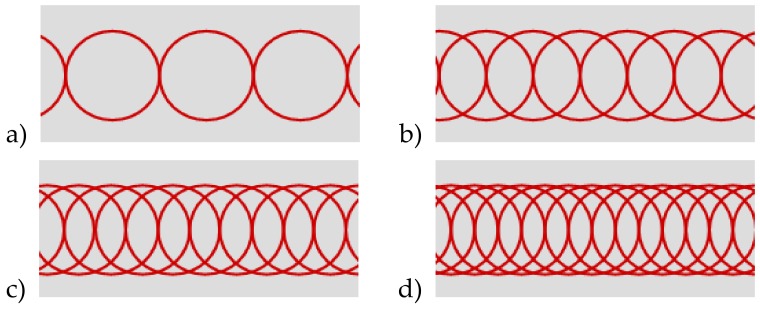
Pulse overlapping: (**a**) 0%; (**b**) 50%; (**c**) 67%; (**d**) 75%.

The laser beam was delivered to a galvanometer scanner head, and a 163 mm F-Theta lens was used to focus the laser beam (Figure 2). The focused spot is about 21µm. A hatching technique was used to machine over an area. The technique consists of filling a defined shape with lines, and it removes the material from the processed area (Figure 3). If the lines’ distance is close to the spot size or smaller, it is possible to achieve deep engraved structures (Figure 4). 

In the results described the follow parameters were used:

- Average power: 5–12 W
- Pulse frequency: 50 kHz
- Scanning speed was tested in the range of 50–1000 mm/s, which affects pulse overlapping along a single line (Figure 4) within a range of 0% to 90%
- Hatching distance between parallel lines was between 5 and 20 μm
- Hatching direction was always bidirectional with two angles: 0^o^ and 90^o^ (Figure 3)

During the laser machining of materials, no purge gas was used. 

The microstructures produced were examined with confocal microscopy, scanning electron microscopy (SEM) and Raman Spectroscopy. Confocal laser scanning microscope (CLSM), Nikon MA200 (Tokyo, Japan), was used to examine the geometries and the roughness of manufactured mictrostructures. The obtained results concerning the geometry were confirmed by SEM (S-3000N Hitachi, Tokyo, Japan).

Raman spectra were recorded using the confocal Raman microspectrometer T-64000 (Jobin–Yvon, Lille, France), equipped with the microscope BX-40 (Olympus, Tokyo, Japan). The 514.5 nm Ar line was used for sample excitation. The other parameters of spectra acquisition (time, laser power) were adjusted to obtain good quality spectra. The diameter of the laser beam was 1.5 μm, and the light intensity across the beam was of Gaussian distribution.

## 3. Results and Discussion

**Figure 5:**
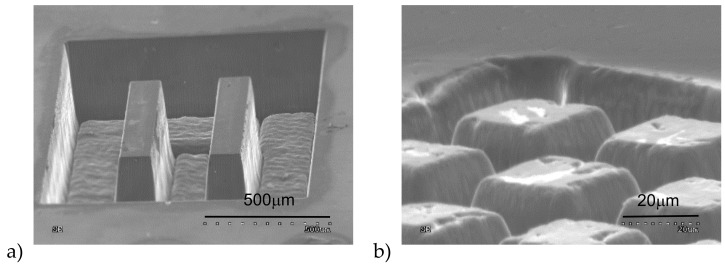
Examples of SEM images of different structures obtained on the surface of CVD diamond plate at laser system condition: average power—9.5 W, scanning speed—250 mm/s, hatching distance —10 and 20 μm (image a and b, respectively).

Micromachining formation of the chemical vapor deposition (CVD) diamond surface was performed, with the application of three selected laser powers and different parameters of surface scan by laser. At the beginning, we focused on finding the best parameters to cut diamond and next to form different structures on the surface of diamond plate. Results of this investigations are shown in Figure 5.

**Figure 6:**
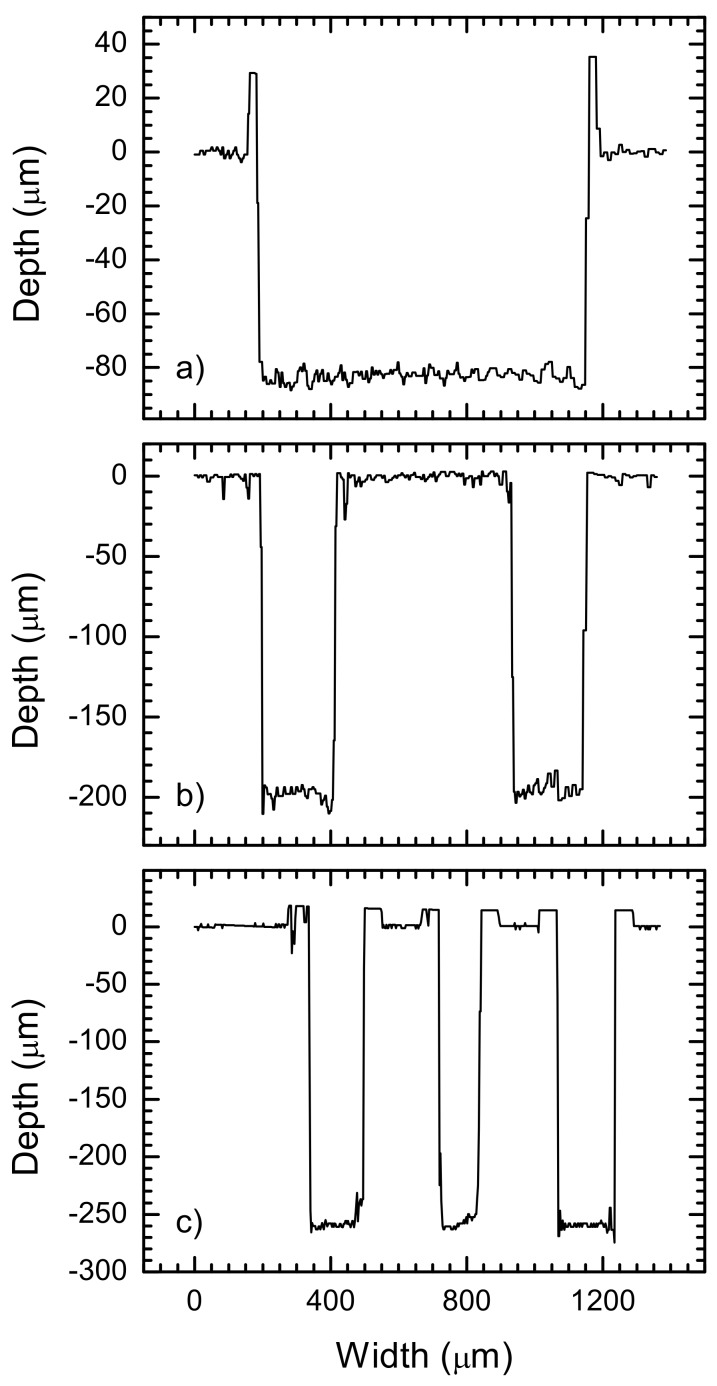
Profiles of select places after laser formation obtained by confocal microscope for micromachining process with scanning speed—100 mm/s, hatching distance—10 μm (**a**) and 5 μm (**b** and **c**), average power—9.5 W, (**a** and **b**) and 5 W (**c**).

Examinations of modified places of diamond by confocal microscope show that narrow—12.8 μm, and deep—245 μm, grooves can be obtained. The slope of the groove wall in all cases was no lower than 88.9^o^. An example of microstructures’ profiles after micromachining treatment by laser is presented in Figure 6. A detailed analysis of these places shows that modification results depend on parameters of laser treatment—laser power, scan grid and speed during micromachining operation. Small hillocks form on groove edges, constituting diamond surface debris. The maximal value of the height of this hillock—about 70 μm, was obtained at 11 W of average power (maximal during the surface formation) and low scan speed—50 mm/s. In Figure 6, the height of the hillock on the edge of the grooves does not exceed the value of 40 μm. The existence of these hillocks can be explained by a lack of purge by gas during the laser formation process of the diamond surface. The size of the hillocks is affected by the condition of laser processing.

**Figure 7:**
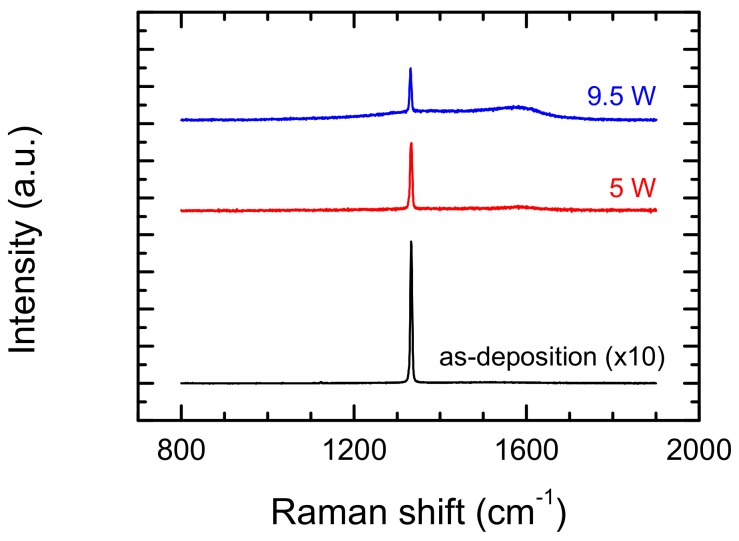
Effect of laser power on Raman spectra from modified surface of polycrystalline diamond. Scan speed during the laser etching process was 100 mm/s, hatching distance—5 μm.

Prior to and after laser formation, the diamond sample was examined by Raman spectroscopy. Obtained results show the wide variety of spectra for the different parameters of laser processing. Figure 7 shows the Raman spectra of the diamond surface (as-deposition) with comparison of two places with manufactured microstructures, manufactured with a 100 mm/s scan speed and 5W and 9.5W of average laser power. Both Raman spectra from the bottom of the groove show a strong diamond peak, which only slightly shifted to lower value with increased power, and was slightly wider in comparison with no modified surface. In conclusion, no modified surface diamond peak is observed at 1332.9 cm^−1^ with FWHM = 4.1 cm^−1^, for 5 W of average power at 1332.6 cm^−1^ with FWHM = 4.8 cm^−1^ and for 9.5 W of average power at 1331.3 cm^−1^ with FWHM = 4.2 cm^−1^. Interning is Raman Spectra for 9.5 W average powers. In these spectra, the peaks assigned to graphite are clearly visible next to the diamond peak.

**Figure 8:**
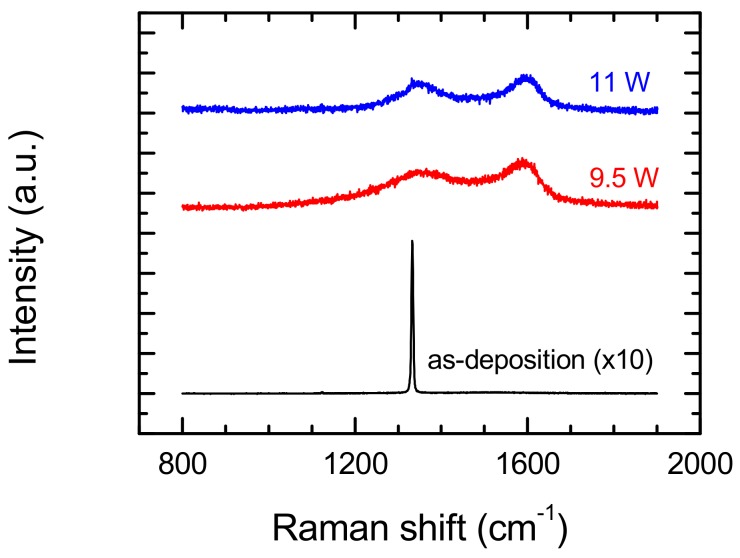
Effect of laser power on Raman spectra from modified surface of polycrystalline diamond. Scan speed during the laser etching process: 400 mm/s, hatching distance—10 μm.

**Figure 9:**
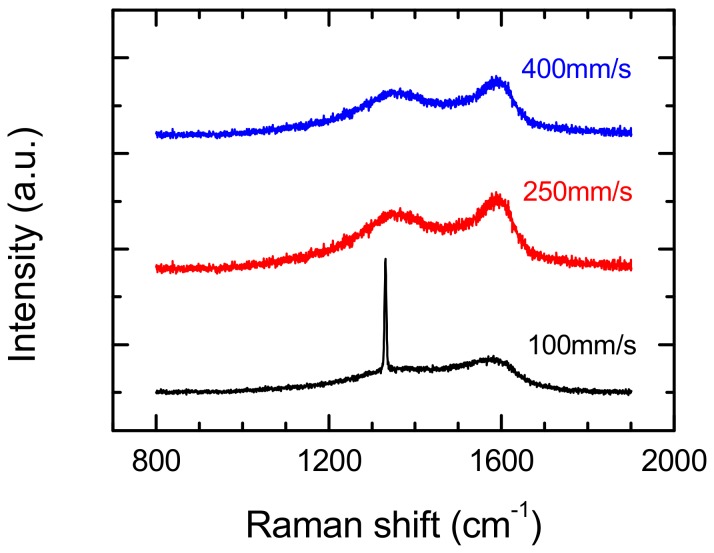
Effect of scan speed on Raman spectra from modified surface of polycrystalline diamond. Laser power during the etching process: 9.5 W of average power, hatching distance—10 μm.

The surface microstructures of the diamond sample were changed completely in the case of micromachining treatments at a scan speed of 400 mm/s and applied average laser power of 9.5 and 11 W. The diamond peak observed prior to modification in Raman spectra completely disappeared at these parameters (Figure 8). In this place, the D and G lines clearly appeared (around 1350 cm^−1^ and 1580 cm^−1^, respectively) to be related to sp^2^ carbon. The comparison of the Raman spectra from Figure 5 and Figure 6 suggests that the microstructures of diamond surfaces after laser treatment are strongly affected by scan speed. There is excellent confirmation of this in Figure 9, where Raman spectra performed at a constant laser average power (9.5 W), and various scan speeds are shown—there is an observed diamond peak (1331.3 cm^−1^) and D and G peaks in the case of spectra obtained at a scan speed of 100 mm/s. With an increase in scan speed, the diamond peak disappears from the spectra and only the D and G peaks are identified, typical for low-quality CVD diamond. Existence of only these peaks in the spectrum can be explained by:

- covering of modified surface by the products of this modification, or
- by the phase transition of surface layer to graphite-like structure [14,20], which is affected by the heating of the surface as a result of a rapid return to the same modified place of laser during rescanning of the surface. A high scan speed increases surface temperature, while a low speed allows for better heat transfer from the modified place—lower surface temperature.

In both cases, identification of a graphite-like structure on the top of a diamond bulk material can be explained by the fact that the Raman cross-section of sp^2^-bonded carbon is 50–100 times higher than that of sp^3^ - carbon for an excitation wavelength of 514 nm [21].

**Figure 10:**
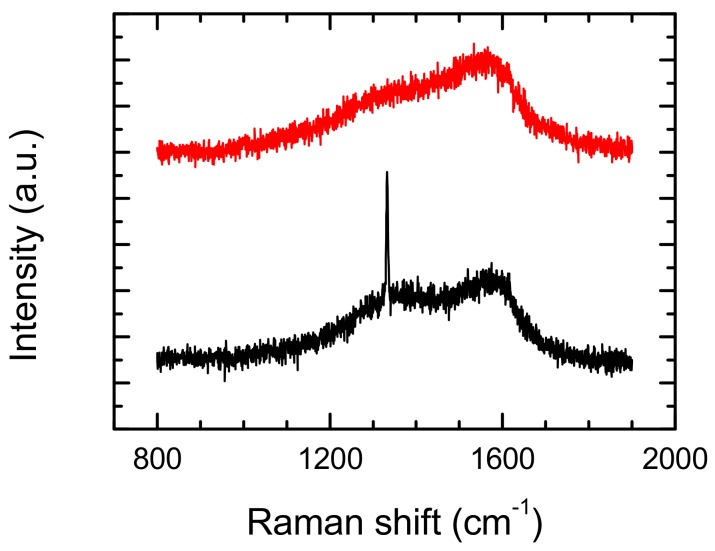
Raman spectra from two places of the laser modified surface at 9.5 W of average power, scan speed 100 mm/s and scan grid 5 × 5 μm. Diamond peak appears only in one spectrum at 1332.5 cm^−1^ with FWHM = 4 cm^−1^.

In the first case, the use of ablated materials results in the interaction of the diamond surface with the impulse of laser light, in the form of graphite-like carbon, covering the bulk diamond surface because no purge gas was used during the laser formation. Nevertheless, the modification of the surface layer was examined by Kononenko et al. [22] They observed the transition of Raman spectra from the included diamond peak at 1332 cm^−1^ to a typical performance for low-quality CVD diamond (only with D and G peaks), after materials transformation by laser at a very short distance from the modified surface. Confirmation of this thesis can be seen in the Raman spectra, shown in Figure 10. Raman spectra was from the same microstructure (the same laser processing parameters), but in two closely lying points of the modified surface. Spectra differ in appearance, and the no-appearance diamond peak was at 1332 cm^−1^. This effect is caused by a scan grid (in this case equal to 5 × 5 μm) used during the shaping of the diamond. Local heating by laser leading to a phase transition and the depth of the transition depend on the scan grid and speed. 

So, the precise microstructures obtained in diamond, both with and without modification of the material, can be obtained. The investigations presented above lead to many new applications, for instance different Microelectromechanical Systems (MEMS) and microfluidic devices.

## 4. Conclusions

According to the presented results, the laser micromachining of the MW PECVD diamond surface allows for the formation of a groove with excellent shape:

- Narrow:     10 μm
- Depth:      270 μm
- Slope:      88^o^
- Roughness (Ra):  0.135 μm

The selection and control of laser parameters can also lead to specific modifications of diamond substrate in chosen places. Some of the Raman spectra in this research show additional peaks, indicating the growing disorder of diamond after the interaction with a laser beam (sometimes amorphous phase appears) or graphitization. The appearance of these peaks, typical for low quality CVD diamond, is strongly affected by scan speed during micromachining laser formation. The rapid return of laser in the same modified place during rescanning of the surface at high scan speed leads to an increased surface temperature, while low speed allows for better heat transfer from the modified place—lower surface temperature; finally, without graphitization, material is obtained.

This concludes, based on investigations, the effect of the laser micromachining process of diamond plate. The best parameters leading to the best geometry of groove are; processing with 9.5 W of laser average power conducted with 100 mm/s scan speed and 5 μm hatching distance. Parameters for the wider groove of 25 μm allow the obtainment of a diamond surface without hillocks on the groove edges, however, the surface has a relatively high roughness (Ra ≥ 0.5 μm). This means that finally, the parameters of the micromachining process have to be changed in order to obtain a smooth surface.
